# Supplementary material for: TNF-α induces Claudin-1 expression in renal tubules in Alport mice
Source: PLoS One. 2022 Mar 10;17(3):e0265081. doi: 10.1371/journal.pone.0265081 (PMC8912176; doi:10.1371/journal.pone.0265081)
Supplement: S1 Method — (DOCX) [file pone.0265081.s001.docx]

**S1 Method. Establishment of Col4a3 knockout mice.** A pair of ZFN mRNAs which target to the exon 48 of mouse Col4a3 were custom-designed by Sigma-Aldrich (target sequence: CCGACACAGTCAAACCACggccatTCCTTCATGCCCTGAA). The uppercase letters present the target sequence for zinc finger nuclease binding and the lowercase letters indicate the cutting site. The exon 48 encodes the first part of the NC1 domain which is a functional domain of the normal collagen chain. A mixture of 2.5 ng/μL of each ZFN mRNA and 1 μM ssODN as donor template to insert a terminal codon at the target site were microinjected into the pronuclei of C57BL/6J mouse fertilized eggs. Embryos were transferred to the oviducts of the pseudo-pregnant Jcl:ICR female mice (CLEA Japan, Tokyo). Offspring were screened for mutations at around the target site of the Col4a3 gene. Genomic PCR was conducted with the following primer set: 128082F (5’-ATCATCTCTGGAAACGCCTTCACAGACACG-3’) and 128723R (5’-ATGGTGAATCTAGCTGCATTTCCTCTAGC-3’). The PCR products were sequence-analyzed using the primer 128106F (5’-ACACGCCTAGGTATGTCTTACTAGTCTCC-3’) to determine the mutations (Fig1B, Supplemental Fig1A & 1B). As a result of genotyping, insertion of the ssODN was not found but several indel mutations were successfully obtained. First, we crossed these mutant mice to obtain several bi-allelic mutants because these indel mutations in the exon 48 were expected to result in premature terminal codons because of frame shifts around the target site. As we expected, no apparent phenotypic differences were observed in these Col4a3 mutant mice (data not shown). Therefore, among these indels, 5 nucleotides (ACGGC) were deleted to establish a knockout mouse strain (Fig1B, 1C) and was backcrossed to C57BL/6N mice for at least 10 generations to reduce the risk of possible off-target mutations by ZFNs. After backcrossing, heterozygous knockout mice were intercrossed to obtain the homozygotes. After several assessments, no gross abnormalities other than renal failure were observed in the homozygotes, indicating that the ZFNs caused few or no off-target effects in our mouse strain.
