# Supplementary figures and images for: TNF-α induces Claudin-1 expression in renal tubules in Alport mice
Source: PLoS One. 2022 Mar 10;17(3):e0265081. doi: 10.1371/journal.pone.0265081 (PMC8912176; doi:10.1371/journal.pone.0265081)

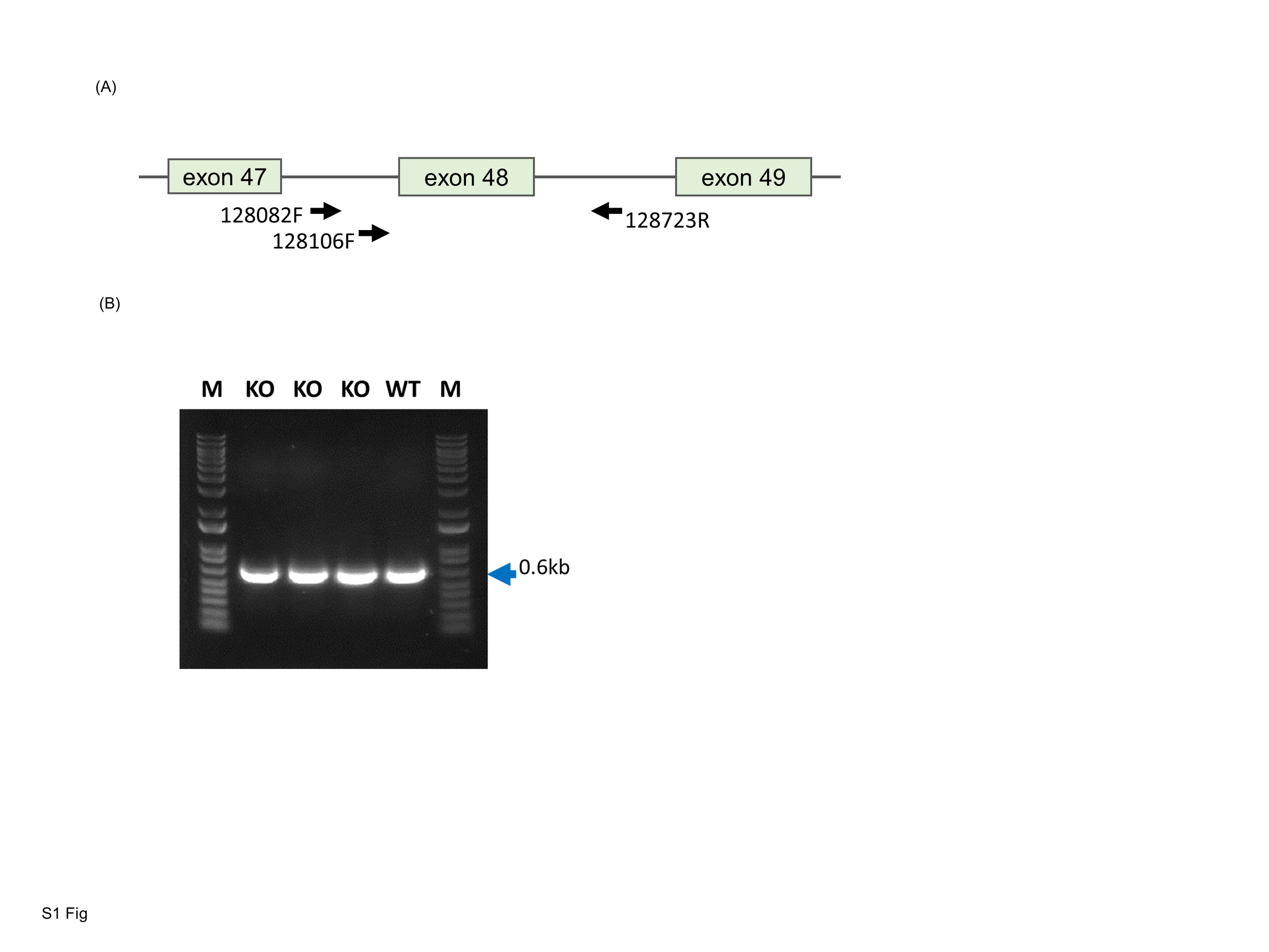

Supplement: S1 Fig — (A) Schematic representation of the genomic structure of the exons 47 to 49 of mouse Col4a3 gene. Arrows show the location of primers used for PCR reaction followed by sequence analysis. (B) Representative agarose gel electrophoresis of the PCR product. Specific amplification around the exon 48 of mouse Col4a3 was confirmed by a single band at approximately 0.6 kb. (TIF) [file pone.0265081.s002.tif]

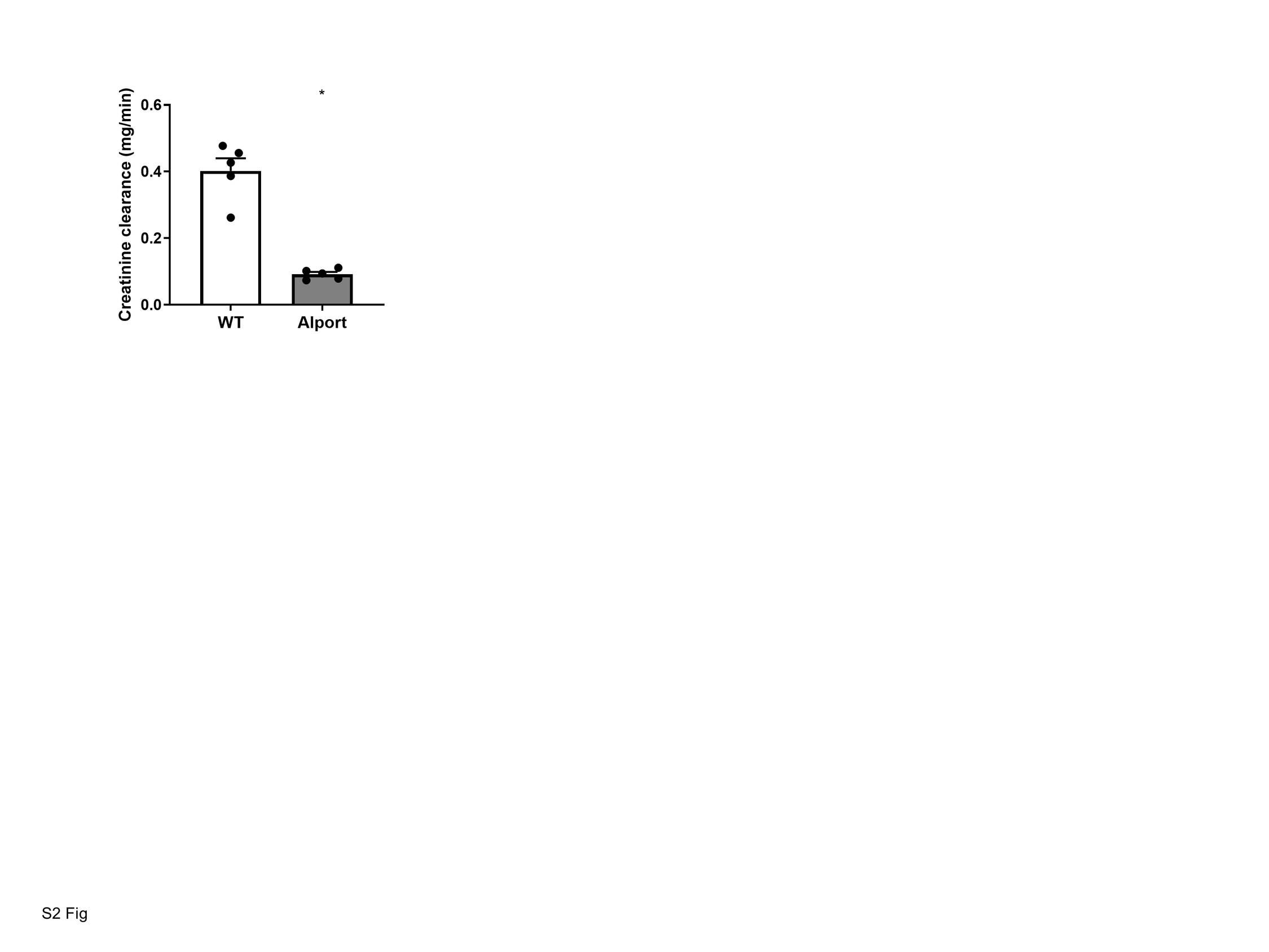

Supplement: S2 Fig — Creatinine clearance was declined in Alport mice at 20 weeks old. The actual values are represented as dots and the columns represent mean + SE. *p<0.05, significant difference versus sham (Student’s t-test). WT: wild type mice, Alport: Alport mice. (TIF) [file pone.0265081.s003.tif]

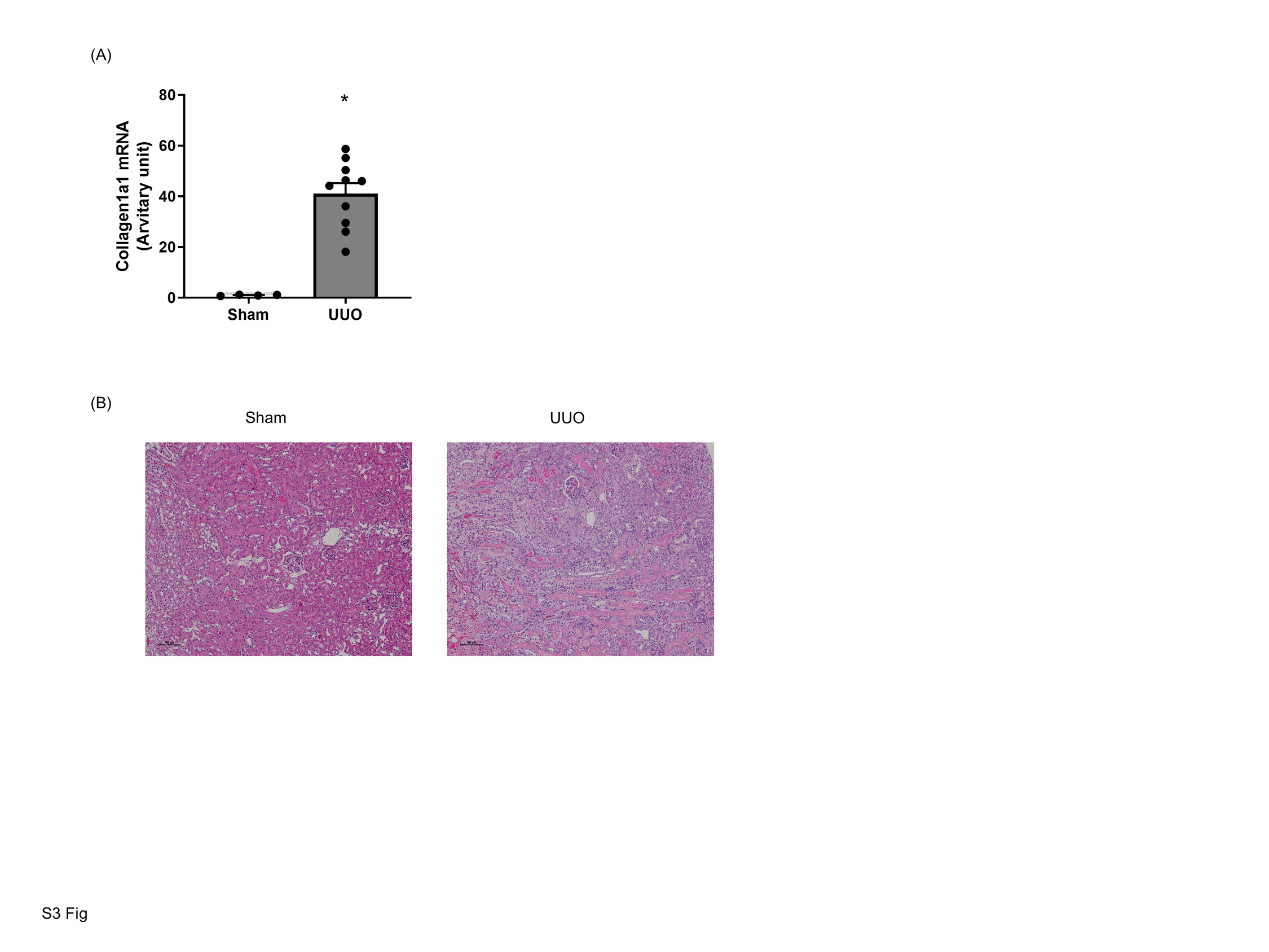

Supplement: S3 Fig — The mRNA expression level of collagen 1a1 was significantly elevated in UUO mice (A). Representative micrographs showing HE stained kidney sections. Scale bars indicates 100 μm (B). Relative mRNA expression was calculated as the ratio to GAPDH expression level. The actual values are represented as dots and the columns represent mean + SE. *p<0.05, significant difference versus sham (Student’s t-test). Sham: sham operated mice, UUO: UUO mice. (TIF) [file pone.0265081.s004.tif]

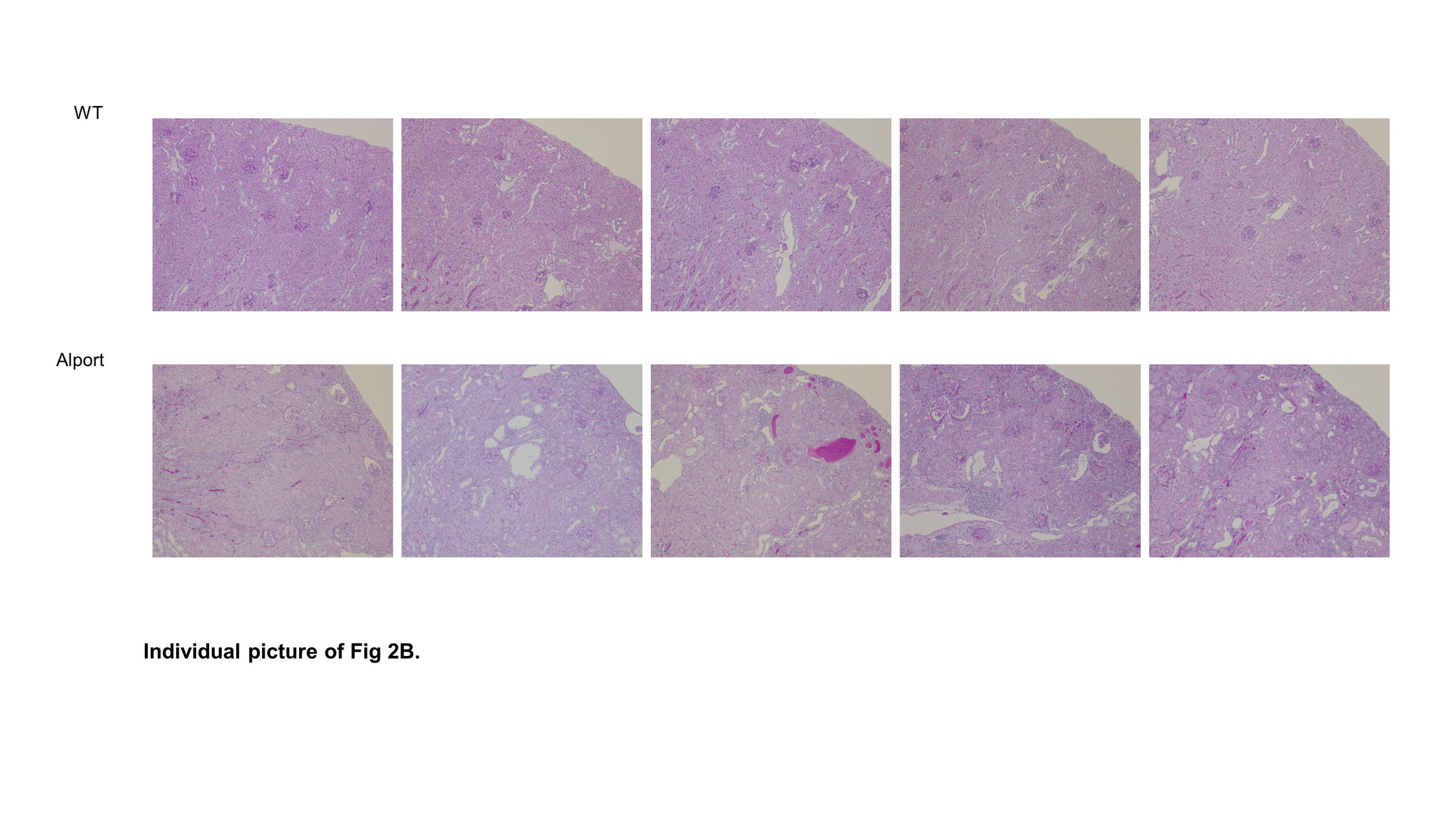

Supplement: S1 Raw image — Individual micrographs showing PAS stained kidney sections. WT: wild type mice, Alport: Alport mice. (TIF) [file pone.0265081.s005.tif]

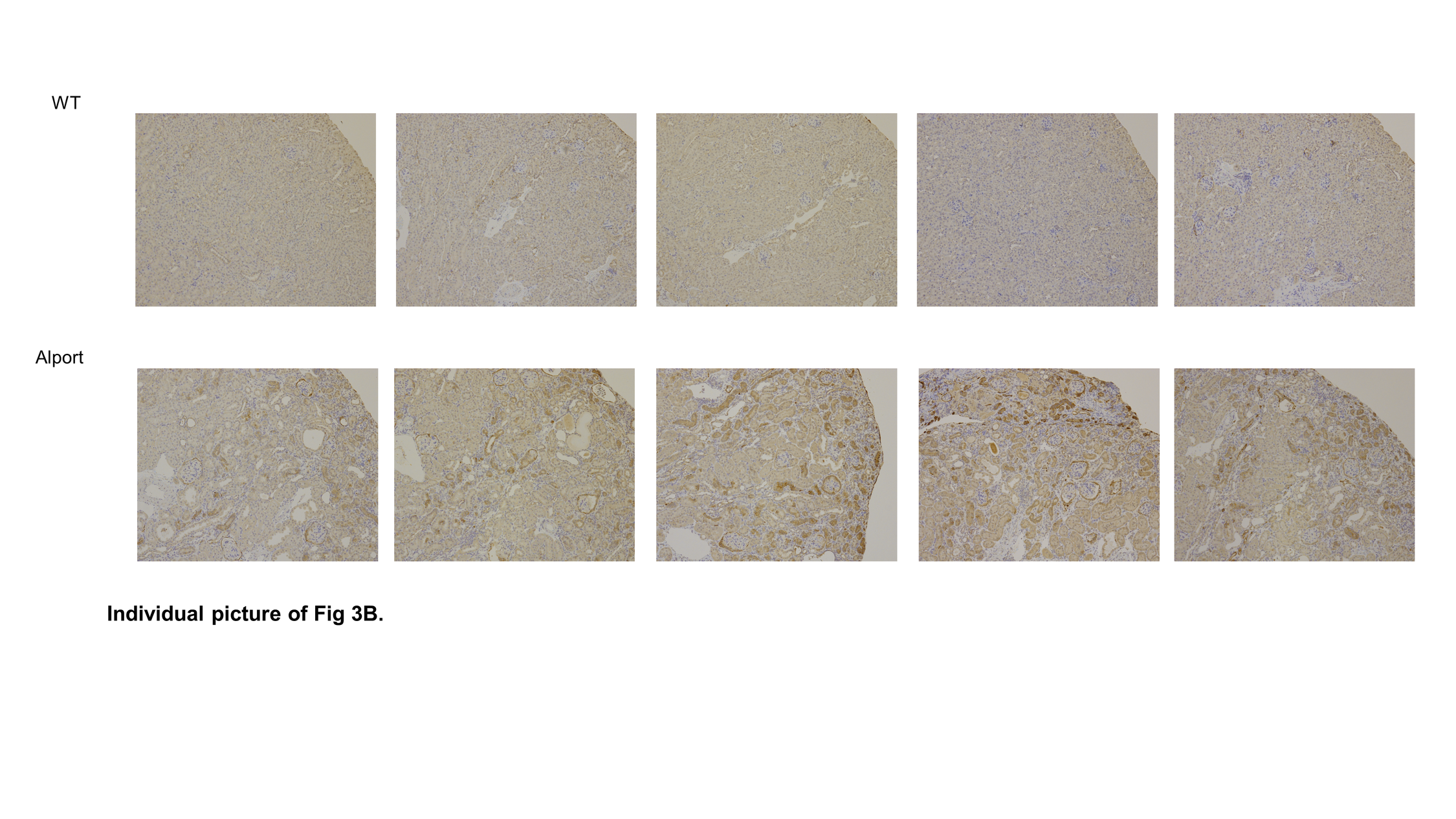

Supplement: S2 Raw image — Immunohistochemical images of CL-1 in the kidney. In Alport mice, CL-1 was strongly stained in proximal tubular cells. WT: wild type mice, Alport: Alport mice. (TIF) [file pone.0265081.s006.tif]

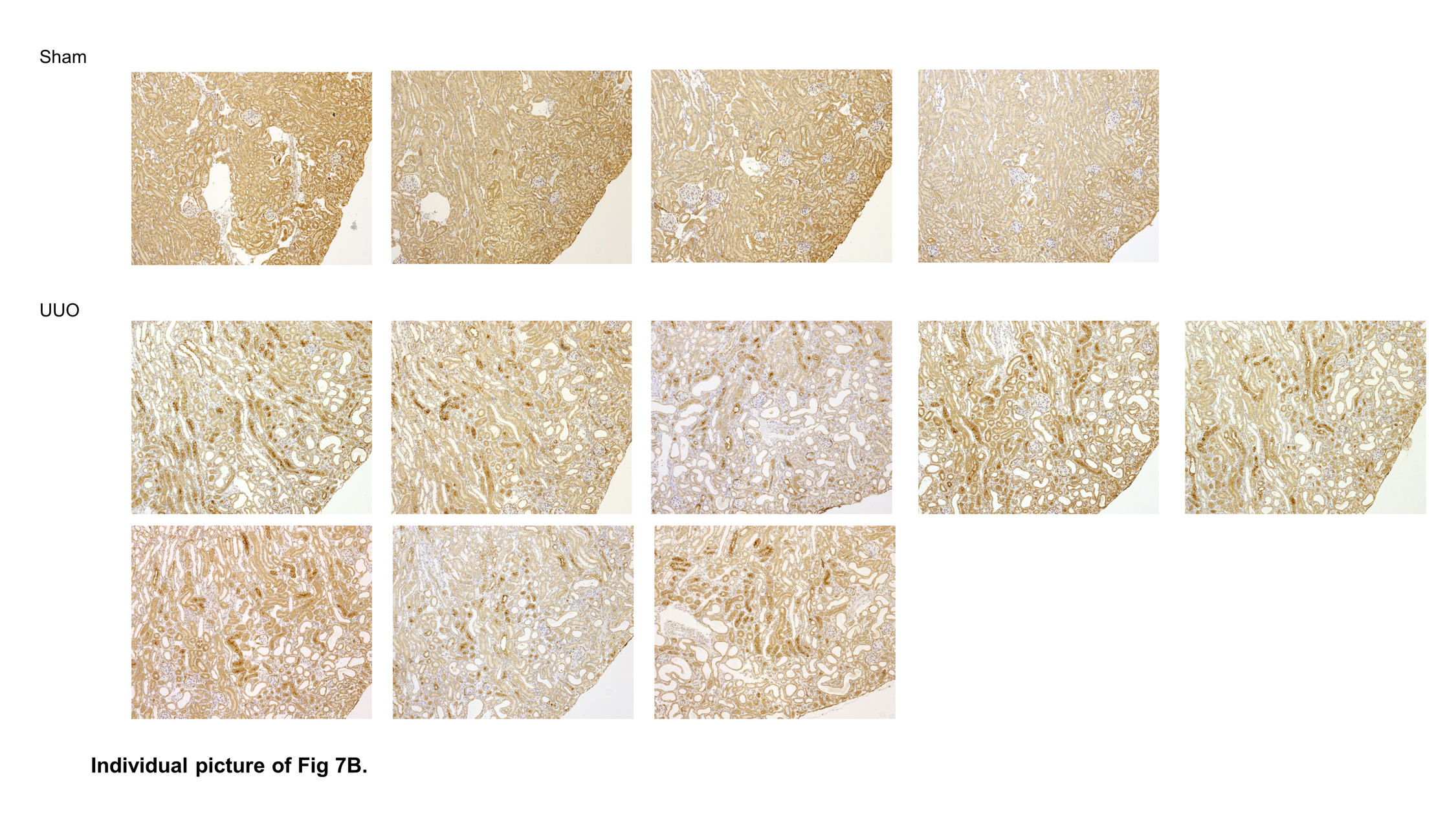

Supplement: S3 Raw image — Immunohistochemical images of CL-1 in the kidney of UUO mice. CL-1 was strongly stained in proximal tubular cells. Sham: sham operated mice, UUO: UUO mice. (TIF) [file pone.0265081.s007.tif]

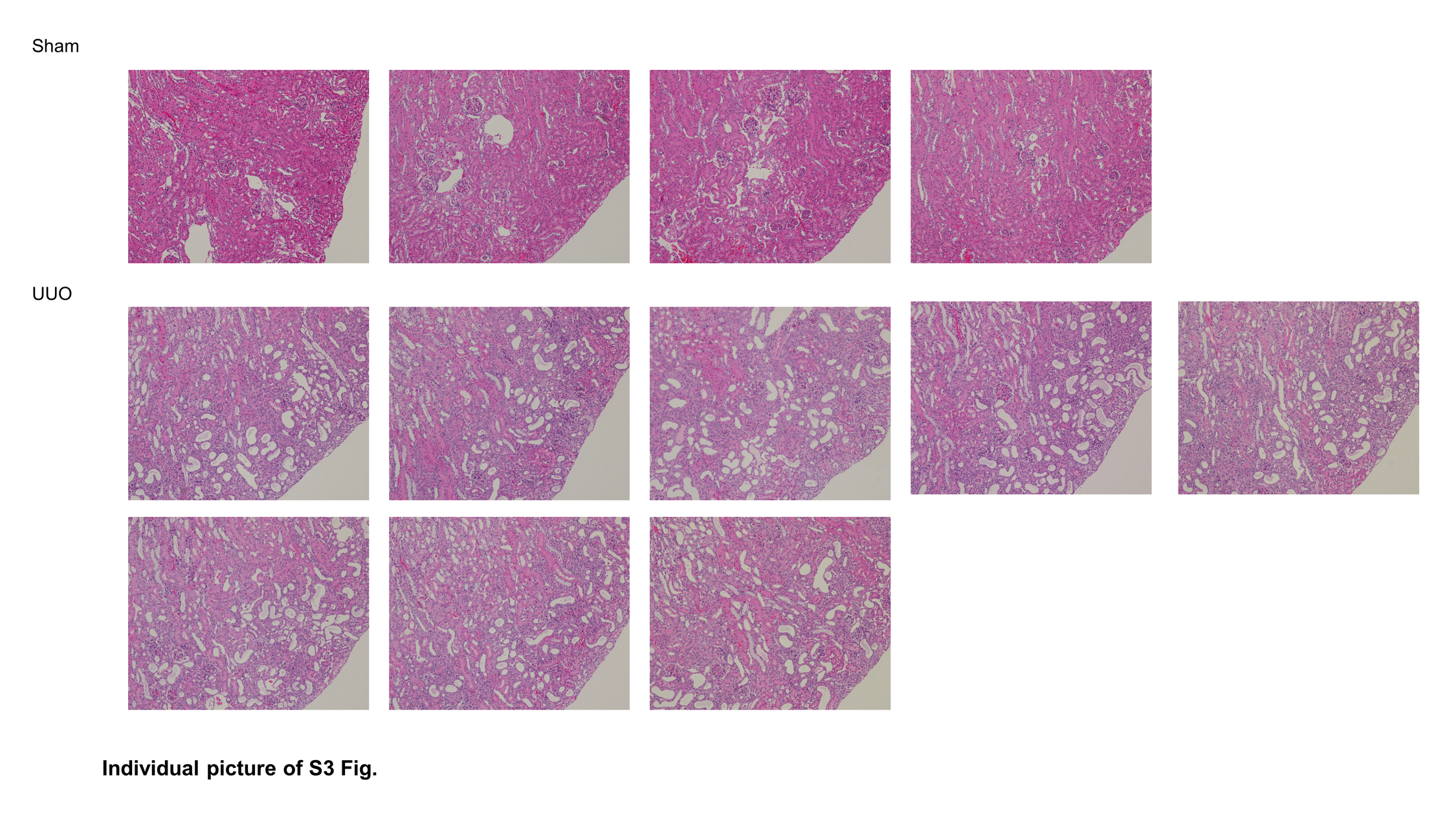

Supplement: S4 Raw image — Micrographs showing HE stained kidney sections. Sham: sham operated mice, UUO: UUO mice. (TIF) [file pone.0265081.s008.tif]

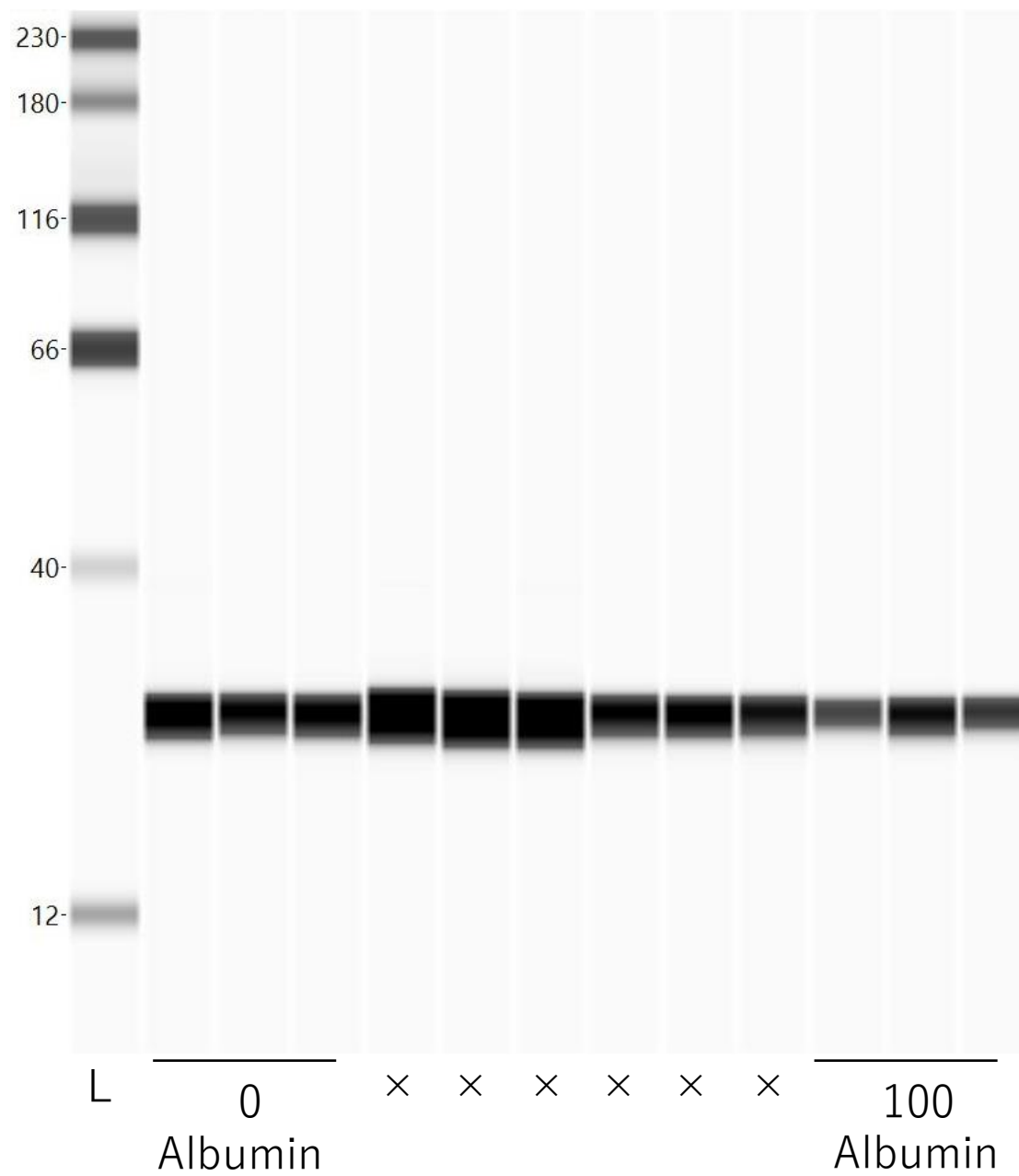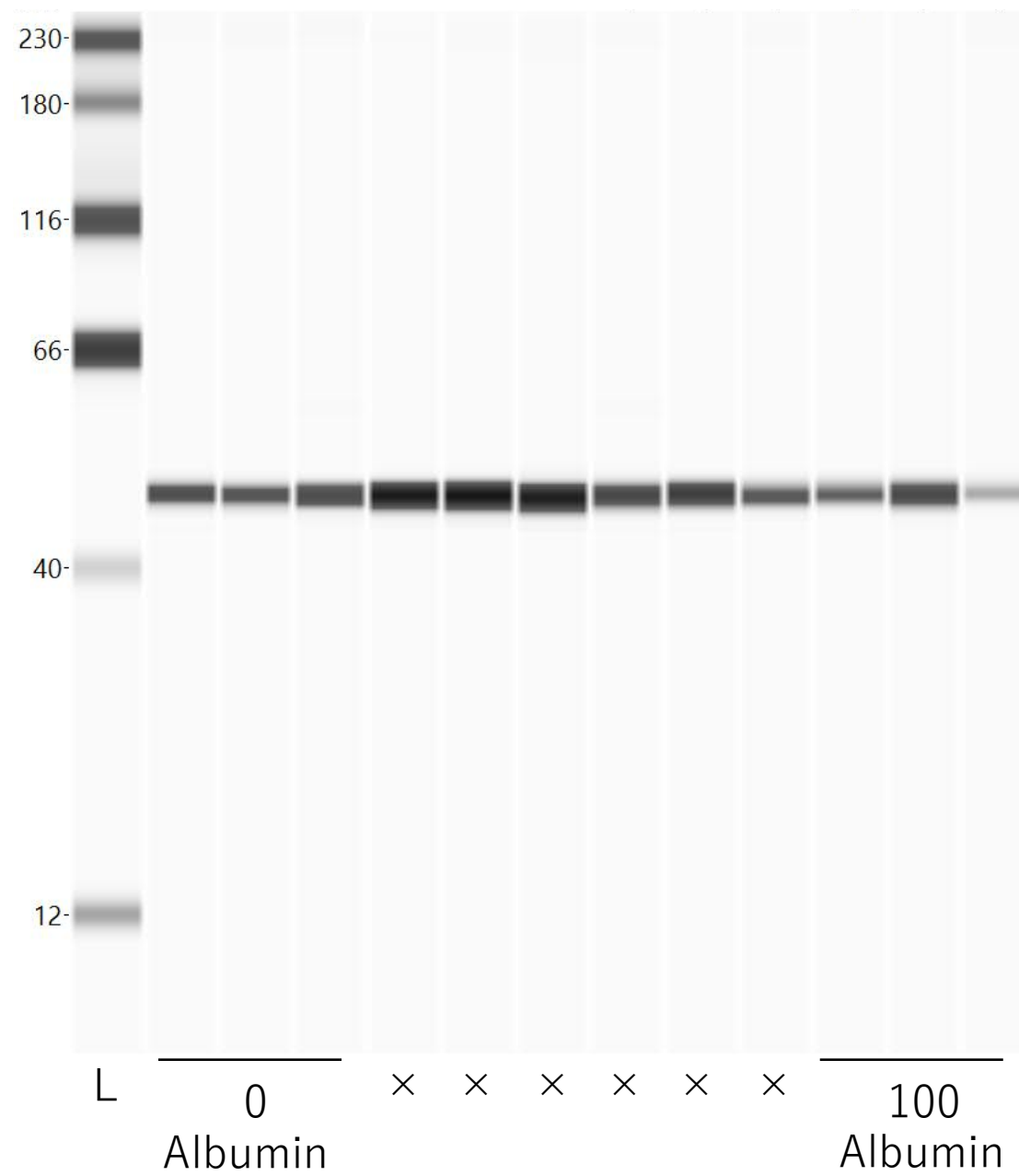

Fig. 5\_raw\_images

L: Ladder

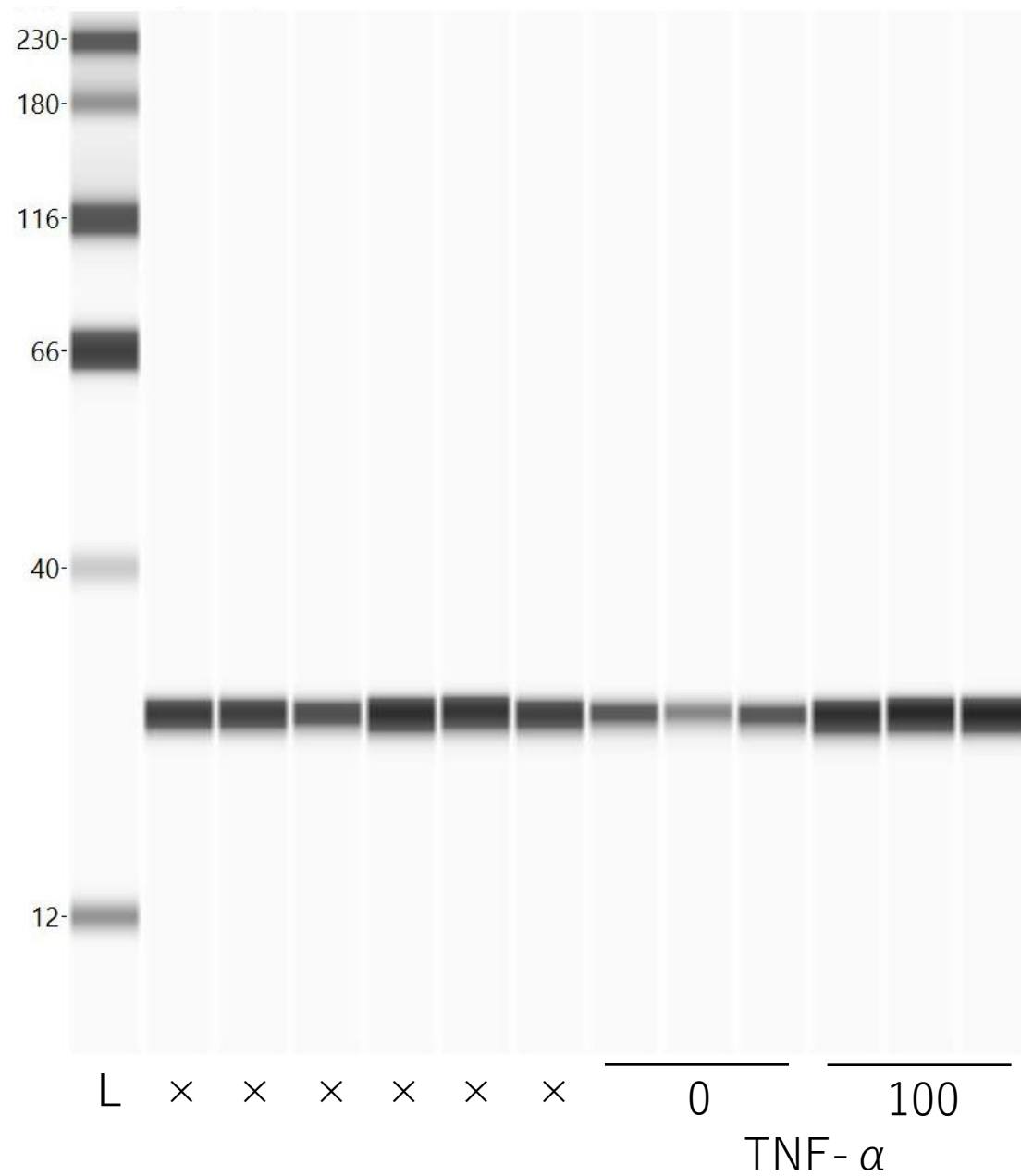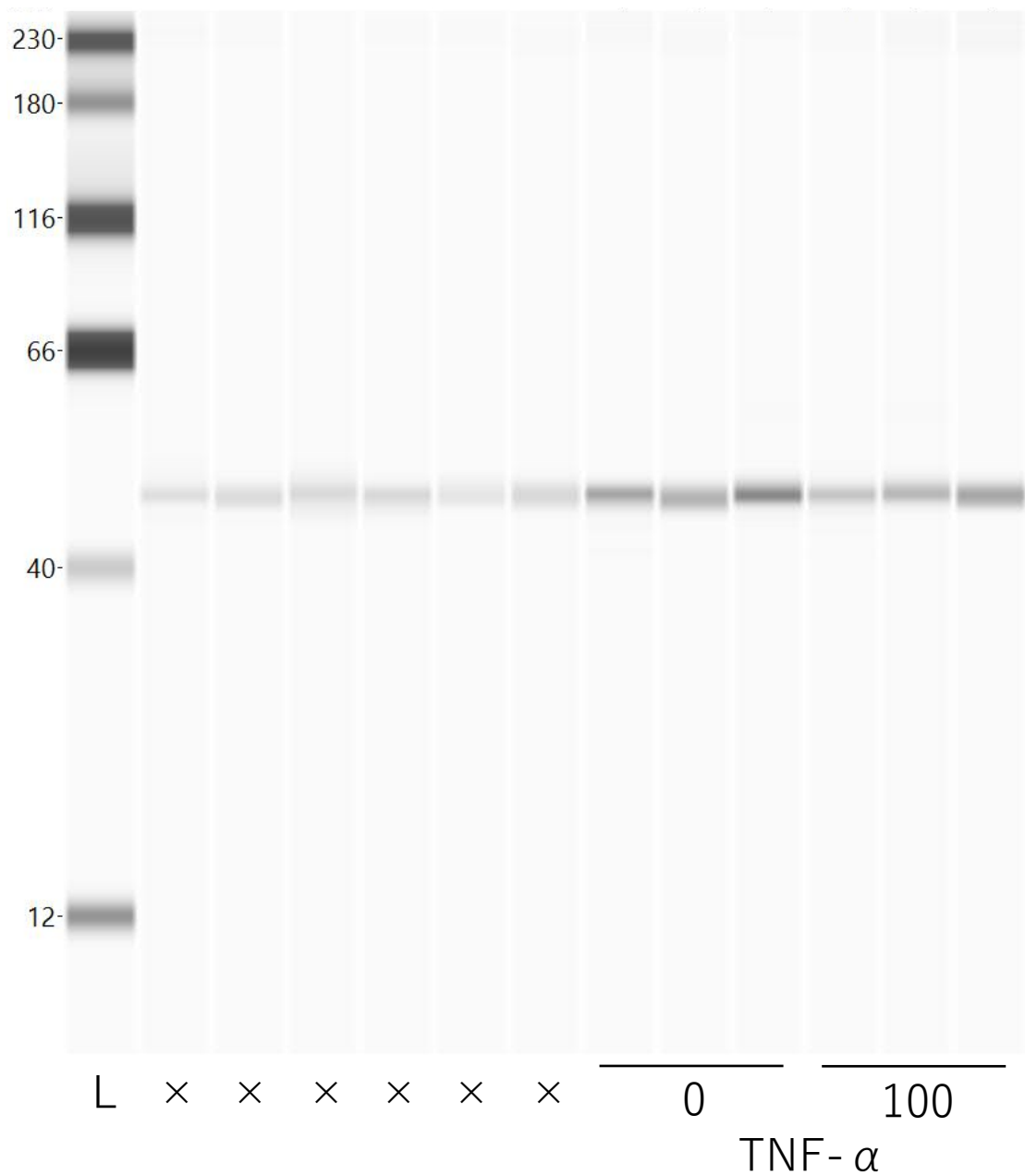

Fig. 6\_raw\_images

L: Ladder

Supplement: S5 Raw image — (PDF) [file pone.0265081.s009.pdf]
