# Supplementary material for: TNF-α induces Claudin-1 expression in renal tubules in Alport mice
Source: PLoS One. 2022 Mar 10;17(3):e0265081. doi: 10.1371/journal.pone.0265081 (PMC8912176; doi:10.1371/journal.pone.0265081)
Supplement: Attachment — Submitted filename: Response letter to reviewer2.docx [file pone.0265081.s011.docx]

Response letter to reviewer #2

Dear reviewer,

Thank you for critically reviewing our manuscript. In response to your comments, we have revised our manuscript as follows.

Minor comments:

1. The title needs some modification as it does not fit well with the study.

According to your suggestion, we changed the title as follows:

Line 2 (Title): TNF-α induces Claudin-1 expression in renal tubules in Alport mice

2.	The western blot images need a lighter exposure to make the changes visible.

According to your suggestion, we lightened the exposure in the Western blot images (Figure 5B, 6B).

3. In figure 4C, the authors have shown increased IFN-γ mRNA in Alport mouse model but have not anywhere in the manuscript its role in changing CL-1 expression as this could also increase CL-1 expression being a pro-inflammatory cytokine.

Although IFN-γ-mRNA tended to increase as you pointed out, the increase was not statistically significant. We could not conclude that IFN-γ mRNA really increased in our study. Therefore, we just focused on TNF-α. We should do further experiments to determine whether IFN-γ is really involved in the changing CL-1 expression.

4. I want authors to show the changes in other claudins, not only claudin 2 (Figure 4B), as many other claudins are involved in inflammatory conditions.

Figure 4B shows the change of CCL-2 (C-C Motif Chemokine Ligand 2), not claudin-2. We apologize for using abbreviation without explanation. We spelled out CCL-2 in the manuscript.

Line 204: C-C motif chemokine ligand 2 (ccl2)

By the way, as you pointed out, other claudins would be involved in inflammation. In the preliminary study using Alport mice we measured several claudins (CL-1, 2, 3, 4, 7, 10, and 17) which are reported to be in the kidney. Although CL-3, -4, and -7 mRNA also significantly increased, the increase in CL-1 mRNA was much more apparent than other claudins. So we focused on CL-1 in this study. However, we would like to further analyze CL-3, -4, or -7 in the near future.

Following data are from preliminary study

5. Under the results section (Expression of CL-1 protein in IgAN patients), lines 275, 276 and 277, the authors have written the following sentences “The Results section should describe the most important findings of the study, analysis, or experiment. The most important results should be indicated, and relevant trends and patterns should be described” which I could not understand and seems a copy-paste from somewhere.

We apologize for the mistake. We deleted these sentences.

6. The authors suggested that the increase in CL-1 expression is caused by inflammatory cells around the renal tubule but have not shown what kind of inflammatory cells anywhere in the study. These results need to be shown as the whole paper is based on inflammation.

According to your suggestion, we added the following words to the Results section.

Line 191: infiltration of inflammatory cells, especially mononuclear cells

Line 203: mononuclear cells

7. The discussion needs rewriting as some of the statements contradict their findings.

According to your suggestion, we rewrote the Discussion e.g.,

Line 307: Recently, injury- induced intratubular EMT has been reported to induce tubular atrophy and kidney fibrosis [31]. Therefore, the inhibition of EMT through the suppression of CL-1 expression may be a potential therapy target for kidney disease.

Line 310: Though we revealed that CL-1 expression changes in diseased kidney, we could not determine whether CL-1 acts as a protective factor or a disease inducing factor in diseased tubules in the present study. To clarify this, we need to evaluate the effect of CL-1 suppression in kidney disease using siRNA or tubule-specific CL-1 conditional KO mice.

8. The paper needs major editing for English and grammatical errors.

Thank you for the suggestion. The manuscript was proofread and edited again by a native English speaker.

We hope that the revised manuscript addresses your concerns.

Yours sincerely,

Naoshi Horiba
